# Evaluating anemia in HIV-infected patients using chest CT

**DOI:** 10.1515/med-2024-0996

**Published:** 2024-07-11

**Authors:** Le Zhang, Yan Bi, Min Qi, Xu-Wen Fu, Jia-Lu Wei, Wei Gan, Long Zhu, Xiang Li, Jin-Song Bai

**Affiliations:** Department of ICU, Kunming Third People’s Hospital/Yunnan Clinical Medical Center for Infectious Diseases, Kunming, 650041, Yunnan, China; Department of Radiology, The People’s Hospital of Lincang, Lincang, 677000, China; Department of Radiology, Kunming Third People’s Hospital/Yunnan Clinical Medical Center for Infectious Diseases, Kunming, 650041, Yunnan, China; Department of AIDS, Kunming Third People’s Hospital/Yunnan Clinical Medical Center for Infectious Diseases, No. 319, Wujing Road, Guandu District, Kunming, 650041, Yunnan, China

**Keywords:** HIV, anemia, computed tomography

## Abstract

**Objective:**

The aim of this study was to investigate the role of the Hounsfield unit value of chest CT non-contrast enhanced scan in evaluating the severity of anemia in HIV-infected patients.

**Methods:**

Patients with HIV infection combined with anemia admitted to the Kunming Third People’s Hospital were retrospectively collected and divided into mild anemia, moderate anemia, and severe anemia groups by peripheral hemoglobin (HB) content and calculated the ratio of ventricular septum density (VSD) to left ventricular density (LVD) and VSD to right ventricular density (RVD); then, the above patients were divided into the critical value group and the non-critical value group according to HB and compared the differences of LVD, RVD, VSD/LVD, and VSD/RVD in the two groups of patients.

**Results:**

A total of 126 patients were included, with a mean age of 47.9 ± 11.1 years; 43 cases were in the mild anemia group, 59 cases were in the moderate anemia group, and 24 cases were in the severe anemia group; the differences in LVD, RVD, VSD/LVD, and VSD/RVD were significant in the three groups; VSD/LVD was an independent predictor for the diagnosis of anemia critical value in the non-critical value group vs critical value group by multifactorial binary logistic regression analysis, and the ROC was plotted using VSD/LVD with an area under the curve of 0.731.

**Conclusions:**

The measurement of cardiac cavity density and ventricular septal density under CT plain film scan has a high accuracy in evaluating the severity of anemia in patients with HIV infection and can quickly determine the severity of HIV infection in the early stage and treat it as soon as possible.

## 1 Introduction

AIDS, or acquired immunodeficiency syndrome, is a chronic infectious disease. It is caused by the human immunodeficiency virus, also called HIV. By 2022, there are 39 million people living with AIDS in the world; 29.8 million people are receiving antiretroviral treatment; 1.3 million new HIV infections; 630,000 people have died of AIDS-related diseases [1]. HIV remains one of the most serious infectious diseases that endanger human health by specifically targeting CD4+ T lymphocytes, a critical component of the human immune system, resulting in a decline in the body’s immune function [2,3,4]. Conclusively, people living with HIV sustain multi-organ and multi-system damage with a wide range of complications.

Anemia is the most frequent hematologic abnormality among people living with HIV worldwide [5,6]. The World Health Organization defines anemia as a hemoglobin (HB) level below 110–120 g/L. Sixty percent of HIV-infected adults in sub-Saharan Africa suffer from anemia, of which 22% are severely anemic [7,8]. In the context of HIV/AIDS, comorbid anemia is clinically common and causes decreased quality of survival and increased mortality in patients. Anemia increases the 1-year mortality rate of HIV-infected patients by about 8 and 55% in severely anemic patients [9,10]. In other words, treatment of anemia improves survival [11]. On the same HIV viral load and CD4+ T-cell count, HIV/AIDS-infected patients with comorbid anemia die at a much higher rate than those without anemia, with decreased mortality after correction of anemia [12]. Comorbid anemia is an independent risk factor for short-term mortality in people living with AIDS [13]. It is reported that 7.2–84% of HIV/AIDS patients are complicated with anemia [5]. Anemia is a multifactorial condition. A study of HIV/AIDS-infected patients with comorbid anemia in Yunnan Province proved that elderly women aged 60 years or above, ethnic minorities, body mass reduction, and initial antiviral regimen using zidovudine are risk factors for comorbid anemia [14]. HIV primarily induces several bone marrow disorders including anemia and thrombocytopenia in peripheral blood, accounting for much of the increased risk of death [15]. For this reason, a monitoring of changes in blood cell counts during HIV infection is a necessity for detecting the development of these blood disorders and for taking the essential clinical interventions to avoid comorbidities [16].

People living with HIV may be at risk for anemia secondary to bleeding from a variety of causes. The origin of the blood is detectable by the physician with the assistance of enhanced CT, such as gastrointestinal bleeding and spleen rupture. The scan then allows for a thorough evaluation of the organs to identify tumors, infections, or other organic lesions that possibly account for the anemia. Enhanced CT can also be of great benefit in visualizing enlarged lymph nodes. Since lymph node lesions may contribute to anemia in AIDS-infected patients as well, utilization of enhanced CT is instrumental in visualizing the size and shape of the lymph nodes as well as the uptake of contrast agent [17]. In conventional chest CT plain scanning, the ventricular density of anemia patients is found to be markedly lower than that the ventricular septum density (VSD) [18]. In this study, a retrospective analysis of adult HIV-infected patients with anemia admitted to our hospital was conducted with a view to exploring the value of measuring cardiac cavity density and VSD under CT plain scanning in assessing the severity of anemia in HIV-infected patients.

## 2 Materials and methods

### 2.1 Subjects

A retrospective study design type was used. From January 1, 2021, to December 31, 2021, 126 adult patients with HIV-infected anemia who underwent chest CT examination were continuously recruited in the Kunming Third People’s Hospital. The inclusion criteria were as follows: (1) patients aged >18 years; (2) those who fulfilled the diagnostic criteria for HIV infection [19], i.e., those who had any of the following: (a) positive HIV antibody screening test and positive HIV supplementary test (positive antibody supplementary test or positive qualitative nucleic acid test or quantitative nucleic acid >5,000 copies/mL), (b) epidemiologic history or AIDS-related clinical manifestations and positive on two HIV nucleic acid tests, and (c) positive HIV isolation test; (3) Laboratory tests showed that HB was less than 120 g/L; and (4) those who were informed and fully cooperated with the present study. The exclusion criteria were as follows: (1) patients aged <18 years; (2) those who were neither able to live on their own nor cooperate in receiving chest CT plain scanning; (3) those whose complete peripheral blood HB level data were not available; (4) those without left ventricular density (LVD), right ventricular density (RVD), VSD/LVD, and VSD/RVD measurements; and (5) those who had other important factors that might affect the study findings, such as comorbidities with other important diseases or complications.

Diagnostic criteria for anemia were as follows [20]: anemia was diagnosed when HB was <120 g/L for adult males, <110 g/L for adult females (non-pregnant), and <100 g/L for pregnant women, which was categorized as mild (>90 g/L), moderate (60–90 g/L), severe (30–59 g/L), and very severe (<30 g/L), respectively, according to HB concentration.

### 2.2 Methods

#### 2.2.1 Peripheral blood HB examination and grouping

Five milliliters of peripheral blood was taken from the patients within 24 h of admission, and their HB levels were measured with a fully automated hematology analyzer (model: Sysmex XN2000, Japan). The patients were divided into the mild anemia group, the moderate anemia group, and the severe anemia group following the diagnostic criteria for anemia and then divided into the critical value group and the non-critical value group according to HB <60 versus ≥60 g/L.

#### 2.2.2 Imaging examination and measurement of ventricular and VSD

Chest CT was performed with GE lightspeed VCT (USA), with a scanning range of thoracic entrance to lung base and a tube voltage of 120 kV; automatic mA technology was used for the tube current, with a scanning layer thickness of 5 mm and a reconstruction layer thickness of 1.25 mm. All patients underwent CT examination within 48 h after peripheral blood HB examination, during which no treatment such as blood transfusion was performed. The LVD, RVD, and VSD of patients were measured in Hounsfield Unit (HU). Three measurements were averaged, and accordingly, the ratio of VSD to LVD and the ratio of VSD to RVD were calculated to test for differences in LVD, RVD, VSD/LVD, and VSD/RVD in the mild, moderate, and severe anemia groups.

#### 2.2.3 Plotting of regions of interests (ROIs) and acquisition of Hounsfield values for CT scan data

Selection of appropriate slices was as follows: CT scan images were opened and slices containing ROIs were selected. Adjustment of window widths and levels of images: window widths and levels were adjusted as needed to optimize the image contrast. ROIs were drawn around the region of interest by OsiriX, a drawing tool, usually rectangular, circular, or hand-drawn, ensuring that all key structures were included.

A CT value (HU) measuring tool was used on the selected ROIs. The tool usually provides parameters such as maximum, minimum, and average CT values, from which the desired parameters were selected for statistical purposes. The CT value parameters of the selected ROIs were recorded, representing the density and tissue characteristics of the region. Quantitative analyses, such as densitometry and tissue classification, were performed on HU values measured by DICOM Viewer. Comparisons of these values were made between different regions of the same patient or different patients to detect anomalies or changes. For serial CT scanning, changes in HU values at adjacent time points were analyzed to comprehend dynamic changes in structure or tissue. For specific study purposes, custom scripts were written and image processing platforms such as Python (using the Pydicom library) were used for analysis. With the above methods and tools, ROIs were effectively plotted and Hounsfield values were obtained for CT scan data by the investigators to provide basic data for further image analysis and research.

### 2.3 Statistical analysis

All patients were categorized into critical value and non-critical value groups as per the criteria of grouping in 1.2.1, and the differences between the two groups were examined for five variables: LVD, RVD, VSD, VSD/LVD, and VSD/RVD; variables that were statistically significant (*P* < 0.05) were screened for binary logistic regression and ROC curves were plotted to calculate the prediction efficiency of the regression equation. Statistical treatment was performed on the data included using SPSS 26.0 statistical software, and the count data were expressed as frequencies (percentages) using the chi-square test. Measurement data were tested for normality and homogeneity of variance, and those that met both were analyzed by one-way analysis of variance, expressed as mean ± standard deviation (*X̄* ± *S*); those that did not were tested nonparametrically, expressed as median (interquartile range) *M* (*Q*), with a statistically significant difference at *P* < 0.05. Binary logistic regression was performed using the forward LR method.


**Informed consent**: All subjects gave informed consent and signed.
**Ethical approval**: The study was conducted in accordance with the principles of the Declaration of Helsinki and was approved by the Ethics Committee of the Kunming Third People’s Hospital for ethical rationality.

## 3 Results

### 3.1 General information

A total of 126 adult HIV-infected patients with anemia were recruited, of whom 74 (58.7%) were males and 52 (41.3%) were females, aged 26–81 years with a mean age of 47.9 ± 11.1 years; mild anemia was found in 43 cases (34.1%), moderate anemia in 59 cases (46.8%), and severe and very severe anemia in 24 cases (19.1%). Comparison of body mass index among the three groups revealed no significant difference (*P* = 0.975) in body mass index between mild anemia (23.04 ± 1.75 kg/m^2^), moderate anemia (22.30 ± 2.07 kg/m^2^), and severe anemia (22.80 ± 2.08 kg/m^2^); the statistics of AIDS duration in the three groups revealed no significant difference (*P* = 0.207) between mild anemia (7.28 ± 3.09 years), moderate anemia (7.29 ± 3.08 years), and severe anemia (7.04 ± 2.51 years). Further study revealed no difference between the three groups in terms of comorbidities such as intestinal infections, pneumocystis pneumonia infection, central nervous system infection, and Kaposi sarcoma (*P* = 0.198), and also in terms of the history of previous major illnesses such as heart, liver, and kidney (*P* = 0.996). To conclude, no statistically significant differences were found between the three groups in terms of baseline data such as gender and age (Table 1), which greatly excludes study confounders.

**Table 1:** Basic patient information [ \[\overline{x}\pm s]\] /*n* (%)]

Group		Mild anemia (*n* = 43)	Moderate anemia (*n* = 59)	Severe anemia (*n* = 24)	*P*
Gender (male)		27(62.8%)	34(57.6%)	13(54.2%)	0.770
Age (years)		48.16 ± 10.33	47.86 ± 11.71	47.50 ± 11.33	0.902
Body mass index (kg/m^2^)		23.04 ± 1.75	22.30 ± 2.07	22.80 ± 2.08	0.975
Course of AIDS (year)		7.28 ± 3.09	7.29 ± 3.08	7.04 ± 2.51	0.207
History of heart, liver and kidney diseases	Yes	22(51.2%)	30(50.8%)	12(50.0%)	0.996
	No	21(48.8%)	29(49.2%)	12(50.0%)	
Comorbidities	No	16(37.2%)	35(59.3%)	10(41.7%)	0.198
	Intestinal infection	8(18.6%)	7(11.9%)	8(33.3%)	
	Pulmonary infection	7(16.3%)	9(15.3%)	3(12.5%)	
	Central nervous system infection	10(23.3%)	7(11.9%)	2(8.3%)	
	Kaposi sarcoma	2(4.7%)	1(1.7%)	1(4.2%)	
Medication	Yes	24(55.8%)	33(55.9%)	14(58.3%)	0.407
	No	19(44.2%)	26(44.1%)	10(41.7%)	

### 3.2 Ventricular density and the ratio of ventricular septum to ventricular density

**Figure 1:**
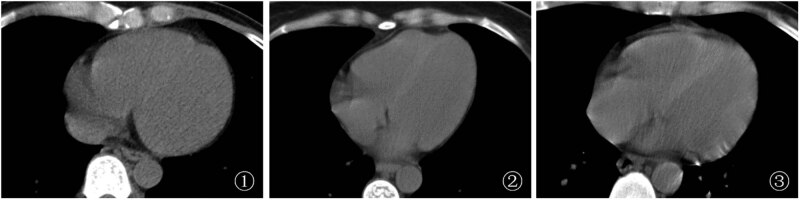
Chest CT images of a HIV-infected patient with anemia (1) A male patient, 44 years old, with mild anemia, HB = 109 g/L, LVD and RVD slightly lower than VSD; (2) A female patient, 42 years old, with moderate anemia, HB = 77 g/L, LVD and RVD lower than VSD; (3) A male patient, 46 years old, with severe anemia, HB = 40 g/L, LVD and RVD significantly lower than VSD.

LVD and RVD of HIV-infected patients with anemia were lower than those of VSD (Figure 1). In the mild anemia group, the mean LVD, RVD, and VSD were 38.90 ± 6.19 HU, 39.74 ± 9.66 HU, and 49.55 ± 6.95, respectively, with a median VSD/LVD of 1.29 (1.20–1.38) and a median VSD/RVD of 1.22 (1.11–1.37); in the moderate anemia group, the mean LVD, RVD, and VSD were 34.54 ± 8.11 HU, 34.99 ± 8.73 HU, and 47.72 ± 7.05, respectively, with a median VSD/LVD of 1.34 (1.26–1.57) and a median VSD/RVD of 1.36 (1.24–1.53); in the severe anemia group, the mean LVD, RVD, and VSD were 32.19 ± 7.23 HU, 34.25 ± 7.92 HU and 49.04 ± 5.98, respectively, with a median VSD/LVD of 1.46 (1.37–1.63) and a median VSD/RVD of 1.40 (1.27–1.6).

LVD and RVD in the mild anemia group were higher than those in the moderate anemia group and the severe anemia group, and VSD/LVD was lower than that in the moderate anemia group and the severe anemia group, with statistically significant differences (*P* < 0.05). VSD/RVD in the mild anemia group was lower than that in the moderate anemia group, with a statistically significant difference (*P* < 0.05); VSD/LVD in the moderate anemia group was lower than that in the severe anemia group, with a statistically significant difference (*P* < 0.05) (Table 2).

**Table 2:** Ventricular density and the ratio of the ventricular septum to ventricular density among the three groups [ \[\overline{x}\pm s]\] /*n* (%)/*M* (*Q*)]

Group	Mild anemia (*n* = 43)	Moderate anemia (*n* = 59)	Severe anemia (*n* = 24)	Mild-moderate		Mild-severe		Moderate-severe	
				*F/Z*	*P*	*F/Z*	*P*	*F/Z*	*P*
LVD	38.90 ± 6.19	34.54 ± 8.11	32.19 ± 7.23	4.35	0.04*	6.70	0.000*	2.35	0.189
RVD	39.74 ± 9.66	34.99 ± 8.73	34.25 ± 7.92	4.74	0.009*	4.80	0.036*	0.063	0.977
VSD	49.55 ± 6.95	47.72 ± 7.05	49.04 ± 5.98	1.30	0.184	0.513	0.769	0.316	0.427
VSD/LVD	1.29 (1.20–1.38)	1.34 (1.26–1.57)	1.46 (1.37–1.63)	−2.805	0.005*	−4.408	0.000*	−2.316	0.021*
VSD/RVD	1.22 (1.11–1.37)	1.36 (1.24–1.53)	1.40 (1.27–1.6)	−2.713	0.007*	−2.723	0.06	−0.619	0.536

* The difference was statistically significant.

### 3.3 Binary logistics regression and ROC curve plotting for the critical value and non-critical value groups

**Figure 2:**
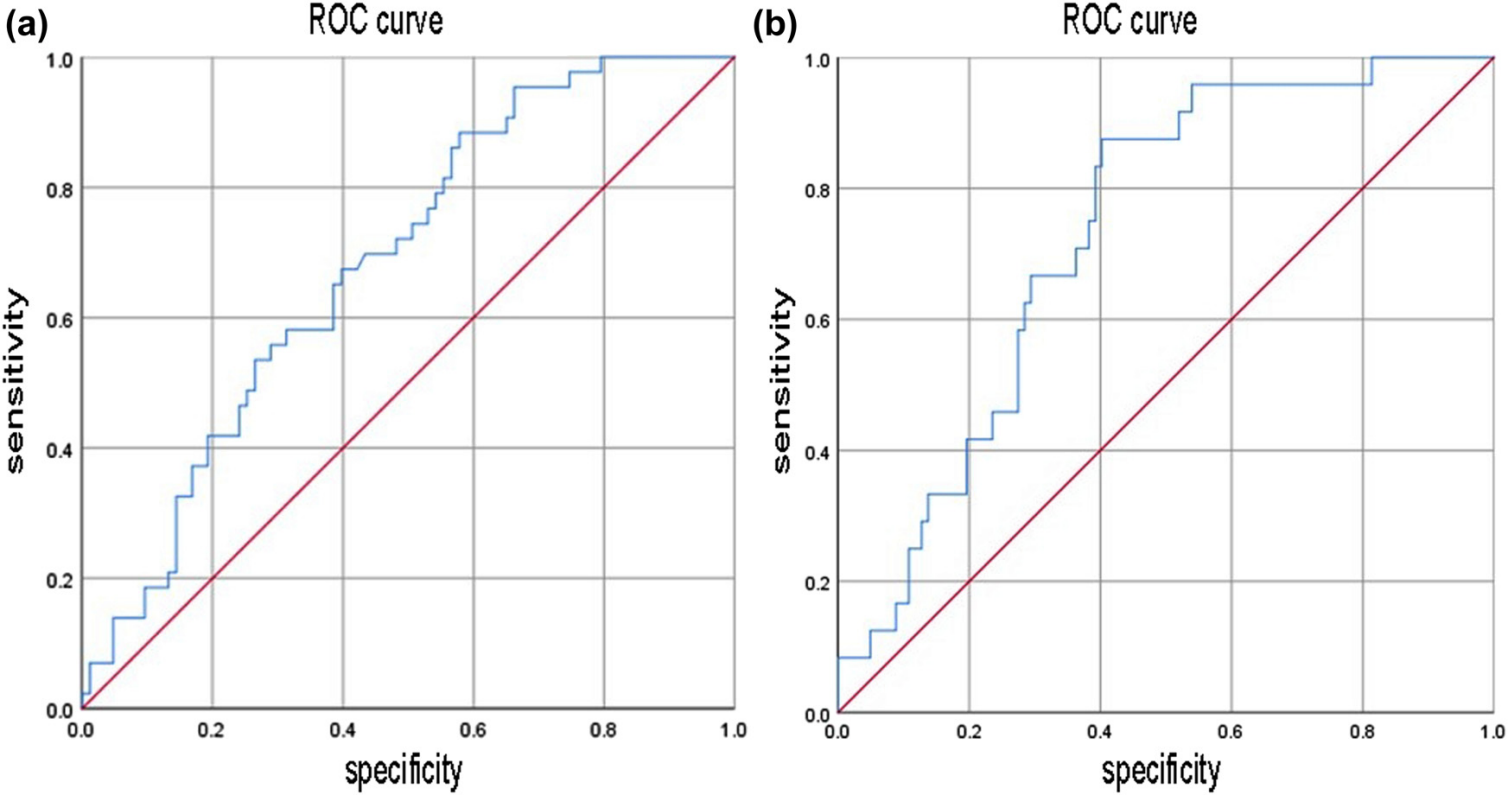
ROC curve of discriminating critical value by probability value of regression equation. (a) ROC curve of LV; (b) ROC curve of VSD/LV.

All patients were divided into the critical value group and the non-critical value group according to HB <60 versus ≥60 g/L, of which 24 cases (19.0%) were in the critical value group and 102 cases (81.0%) in the non-critical value group. There was a statistically significant difference between LVD and VSD/LVD in the two groups (*P* < 0.05) (Table 2). Binary logistic regression analysis was performed with LVD and VSD/LVD as variables, which resulted in the regression equation *y* = −4.721 + 2.249 (VSD/LVD) (Table 3). The ROC curve was plotted by using the probability value of the logistic regression equation. The area under the ROC curve (AUC) of LV was 0.683 [95% confidence interval (CI): 0.587–0.779, *P* < 0.001], and that of VSD/LV was 0.731 (95% CI: 0.635–0.827, *P* < 0.001). At a cutoff value of 1.345, its sensitivity for discriminating the critical value group from the non-critical value group was 87.5% and specificity was 59.8% (Figure 2).

**Table 3:** Binary logistic regression analysis for discriminating the critical value group from the non-critical value group

	*B*	SE	Wald	Df	*P*	OR	95% CI
VSD/LVD	2.249	0.816	7.596	1	0.006	9.481	1.915–46.935
Constant	−4.721	1.227	14.805	1	0	0.009	

## 4 Discussion

In this study, we explored the value of measuring cardiac cavity density and ventricular septal density under a CT plain film scan to evaluate the severity of anemia in patients with HIV infection. The results showed that LVD and RVD in the mild anemia group were higher than those in the moderate anemia group and the severe anemia group, and VSD/LVD was lower than that in the moderate anemia group and the severe anemia group. VSD/RVD in the mild anemia group was lower than that in the moderate anemia group, and VSD/LVD in the moderate anemia group was lower than that in the severe anemia group. Multivariate binary logistic regression analysis showed that VSD/LVD was an independent predictor of the non-critical value group and anemia critical value diagnosis. VSD/LVD was used to draw, and the AUC was 0.731.

Patients with a mean age of 47.9 ± 11.1 years are included in our study, and there is no statistically significant difference in age in anemia severity. So we suggest that anemia should be taken into account in the clinical practice of middle-aged and old-aged HIV/AIDS-infected patients.

Routine blood laboratory examination serves as a valuable tool in the diagnosis of anemia. According to the HB level of peripheral blood, anemia is diagnosed when HB is <120 g/L for males and <110 g/L for females, which is categorized as mild, moderate, and severe according to HB concentration. The technique of testing HB levels in peripheral blood, although accurate and quick, is invasive, resulting in the risk of practice exposure during blood collection by medical staff. HIV infection is prevalent among intravenous drug users within Yunnan Province, and prolonged peripheral intravenous injection further complicates venous blood collection. There are still many challenges in the blood collection of HIV-infected patients: (1) blood routine examination results can be obtained within half an hour to an hour after blood collection. If the results verified by this study can guide clinical work, the results can be obtained by imaging technicians after the scanning is completed (usually 15 s), which attracts the attention and attention of clinicians; (2) blood routine examination results can be obtained within half an hour to an hour after blood collection. Pulmonary is the most common target organ of opportunistic infection. In clinical practice, the vast majority of HIV patients need chest CT examination when they are admitted to hospital, especially emergency patients, which can be evaluated in a short time after admission and have certain benefits; (3) blood routine HIV patients have the risk of exposure to medical staff when nurses collect peripheral blood; and (4) some HIV patients had difficulty in collecting peripheral venous blood due to long-term illness. Timely CT is more expensive than blood routine, but because of the need to diagnose lung inflammation, CT is necessary. Therefore, it is of certain clinical value to evaluate anemia by non-invasive, inevitable, and rapid chest CT.

Iron-containing HB constitutes a major component in the blood that interferes with the attenuation of X-rays [21], and a decrease in its concentration in the blood gradually reduces the CT value. People living with comorbid anemia exhibit reduced blood CT values because of decreased iron-containing HB. They show decreased density of cardiac chambers and chest vessels and relatively increased VSD on CT scans [22], as well as an “aortic ring sign” with a high density of aortic wall and relatively low density of lumen [23]. In the present study of anemia on CT scan images, decreased cardiac chamber density and relatively increased VSD were observed, with some variations for different severities of anemia. Previous studies have revealed the correlation between the measurements of cardiac chamber density and VSD as well as the calculation of their differences and ratios with HB levels in the peripheral blood [24,25] and that the measurements can be evaluated for mild, moderate, and severe anemia [26]. We also demonstrate statistically significant differences in the determination of central chamber density and VSD and the ratio between mild, moderate, and severe anemia in HIV/AIDS-infected patients. However, as noted in some studies, the method may be subject to misdiagnosis due to the imaging of heartbeats and various artifacts. This requires that breathing and movement artifacts should be avoided as much as possible and that CT devices with higher temporal resolution should be selected for practical clinical applications.

In practice, HB in peripheral blood below 60 g/L falls within the range of critical values that should be reported in the laboratory. However, routine blood HB testing is more time-consuming compared to CT examination. In this study, we divided anemia into the critical value group and the non-critical value group according to critical value criteria. The ratio of LV density and ventricular septum to LV density showed statistically significant differences between the two groups. After binary logistic regression analysis of the ratio and plotting the ROC, its AUC was 0.731, and the model had a sensitivity of 87.5% for discriminating anemia status between non-critical and critical values. Despite a relatively low specificity, the higher sensitivity ensures a lower false-negative and enhanced early prompting for clinicians during clinical diagnosis and treatment. Recent years have witnessed the growing importance of artificial intelligence in diagnostic imaging [27], and AI-based organ segmentation techniques are developing rapidly [28]. On the basis of a chest CT plain scan, the technique automatically outlines the entire cardiac chambers and the ventricular septum, which ensures faster prompting of the clinician about the potential anemia status and severity of patients on the basis of previous studies.

There are still some limitations to this study. First, the subjects included are only targeted at people living with HIV infection, for which it is not yet known whether their serum protein levels make a difference in CT values. Second, this is a single-center study with a small sample size and therefore cannot represent a wide range of significance. Moreover, other indexes such as the aorta, pulmonary artery, and inferior vena cava are not included.

## 5 Conclusions

In conclusion, the measurement of cardiac cavity density and ventricular septal density under CT plain film scan has a high accuracy in evaluating the severity of anemia in patients with HIV infection and can quickly determine the severity of HIV infection in the early stage and treat it as soon as possible.
